# Inferring building height from footprint morphology data

**DOI:** 10.1038/s41598-024-66467-2

**Published:** 2024-08-12

**Authors:** Clinton Stipek, Taylor Hauser, Daniel Adams, Justin Epting, Christa Brelsford, Jessica Moehl, Philipe Dias, Jesse Piburn, Robert Stewart

**Affiliations:** 1 Oak Ridge National Laboratory, Oak Ridge, TN 37831 USA; 2 Los Alamos National Laboratory, Los Alamos, NM 87545 USA

**Keywords:** Built environment, Urban planning, Building height, Machine learning, XGBoost, Computer science, Scientific data

## Abstract

As cities continue to grow globally, characterizing the built environment is essential to understanding human populations, projecting energy usage, monitoring urban heat island impacts, preventing environmental degradation, and planning for urban development. Buildings are a key component of the built environment and there is currently a lack of data on building height at the global level. Current methodologies for developing building height models that utilize remote sensing are limited in scale due to the high cost of data acquisition. Other approaches that leverage 2D features are restricted based on the volume of ancillary data necessary to infer height. Here, we find, through a series of experiments covering 74.55 million buildings from the United States, France, and Germany, it is possible, with 95% accuracy, to infer building height within 3 m of the true height using footprint morphology data. Our results show that leveraging individual building footprints can lead to accurate building height predictions while not requiring ancillary data, thus making this method applicable wherever building footprints are available. The finding that it is possible to infer building height from footprint data alone provides researchers a new method to leverage in relation to various applications.

## Introduction

Characterizing urban morphology has grown increasingly important due to projections indicating that global human populations are likely to eclipse 10 billion within the next 50 years^1^, with a disproportionate amount of that growth occurring in cities^2^. As the world becomes increasingly urban, the built environment has undergone rapid expansion^3^. Urbanization contributes to economic growth and development, but also presents broad, multifaceted challenges including environmental degradation, increased pressure on natural resources, carbon dioxide emissions and impacts to public transportation and public health^4–8^.

Knowledge of urban morphology in general, and building height in particular, is imperative for a number of applications including emergency response, sustainable development and the calculation of human population^6,9,10^. Recent research by Schug et al.^10^ has shown that including building height can improve population modeling, especially in regard to studies that only use two-dimensional information or coarse grid sizes. Another study demonstrated that dense urban areas with a high percentage of tall buildings, such as New York City, were the most difficult to accurately estimate population in the absence of building height information^9^.

Another focal area of research on urban morphology is the environmental impact of urban growth on city residents. One specific area of concern is the growth and intensification of urban heat islands (UHIs). UHIs have become more intense globally, and have profound effects on air quality and public health^8,11,12^. As the world’s urban population grows, the importance of UHIs for population heat exposure becomes more significant. Micro-meteorological modeling focused on urban heat is increasing to respond to this challenge, and building height is a critical input to meteorological models such as WRF-UCM^13–15^. Therefore, building height can be integral to helping researchers better understand the current dynamics of the built environment.

**Figure 1:**
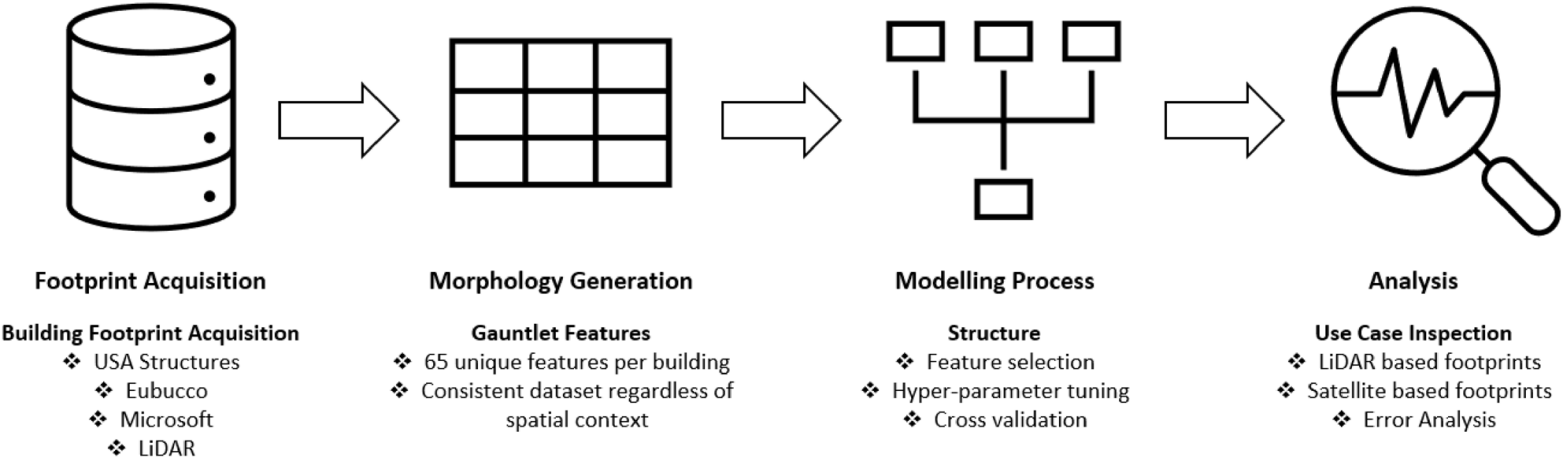
Methodology overview. Each portion of the diagram depicts a significant step in our workflow, starting with footprint acquisition and ending with the analysis. Bulleted points under each process describe in more detail the specific steps in our workflow.

Several decades of research have used remote sensing methods to estimate building height^16,17^. Common practices include the mensuration of height from sensors such as stereoscopic imagery^18–20^, synthetic aperture radar (SAR)^21–23^ and light detection and ranging (lidar)^24–30^. Another common approach leverages sun angles and image metadata to estimate building height by measuring the shadows cast by those buildings^31–35^. Fusion techniques, such as combining laser or radar scans with optical imagery, have also been tested, and in some cases found to be more effective than approaches which rely on a single data source^36–39^. While remote sensing approaches have proven effective in many cases, they do suffer from limitations. For example, in dense urban areas where buildings are closely adjacent, shadows can potentially overlap, which would prevent the successful extraction of individual building heights^40^. Similarly, in such areas, radar layover effects may distort the full reconstruction of buildings and urban landscapes^21^.

Likely as a direct consequence of the high resource cost of remote sensing approaches, the majority of the remote sensing based studies investigated have estimated height in small geographic areas such as at the city level. However, several ambitious projects have recently produced datasets at the national^29,41^, regional^42^, and global level^3,43^. Frantz et al.^41^ developed a 10 m gridded dataset of building height for Germany by employing Sentinel 1 SAR data and Sentinel 2 optical imagery, leveraging building shadows and multi-temporal signatures. Their results showed that the fusion of optical and SAR datasets provided higher accuracy than a single imagery source. Wu et al.^29^ used machine learning with radar, optical and night-time lights data to develop the first ever building height model for China at 10 m. Pesaresi et al.^43^ merged multiple global digital elevation models in conjunction with shadows derived from Sentinel 2 imagery. Using a set of derived features in a linear regression model, the authors estimated building height and volume at the resolution of 250 m × 250 m pixels. The building height and volume datasets created by this work are included in the Global Human Settlement Layer, a suite of products sponsored by the European Commission. Esch et al.^3^ developed a global height product at 90 m spatial resolution, referred to as World Settlement Footprint 3D. This product uses a fusion approach that includes millions of Sentinel 1 and 2 image tiles, as well as data from the German TanDEM-X radar mission. While the data were collected at a higher spatial resolution, the final product has a resolution of 90 m, which potentially limits its application to estimate height at the building level. In addition, the authors in^43^ explicitly outline the scope of their datasets to be used for characterization of different parts of cities and for studies of large urban areas and they explicitly state to not leverage their work for height estimations at the building level.

In contrast to remote sensing methods, researchers have begun leveraging morphology features derived from planar footprint geometries to predict height at the building level^44–46^. There has been rapid growth in the field of morphology features derived from building footprints^47–52^ which have been used across a broad range of studies, including identifying microclimates in China^53^, solar energy potential in the Mediterranean^54^, building occupancy type^55^, UHIs^56^, building age^46^, and local climate zones^57^. The approaches which leverage morphology features to study height at a building-by-building level^44–46^ do not directly measure height, but infer height based on the planar footprint morphology as well as contextual information about the building location, such as road networks^45^. Techniques that leverage machine learning with vector geometries have the potential to be more scalable than remote sensing approaches, since the costs of acquiring and processing imagery and/or point clouds are removed.

While previous work has made great strides in inferring height based on morphology features, the current state-of-the-art machine learning methodology is limited by data availability as it relies on ancillary data^45^. Furthermore, the approaches by Biljecki et al.^44^ and Milojevic-Dupont et al.^45^ are conducted on comparatively costly lidar-derived footprints rather than satellite-derived footprints. The need for ancillary data and lidar footprints can potentially limit the application of machine learning approaches that leverage vector geometries to infer height given the large variability in data completeness across the globe^58^. For example, while there may be high coverage of road networks available in developed countries and near human settlements, such as in the United States (US) or Europe, the availability of quality data in other, less developed areas of the world poses a problem^59,60^.

**Figure 2:**
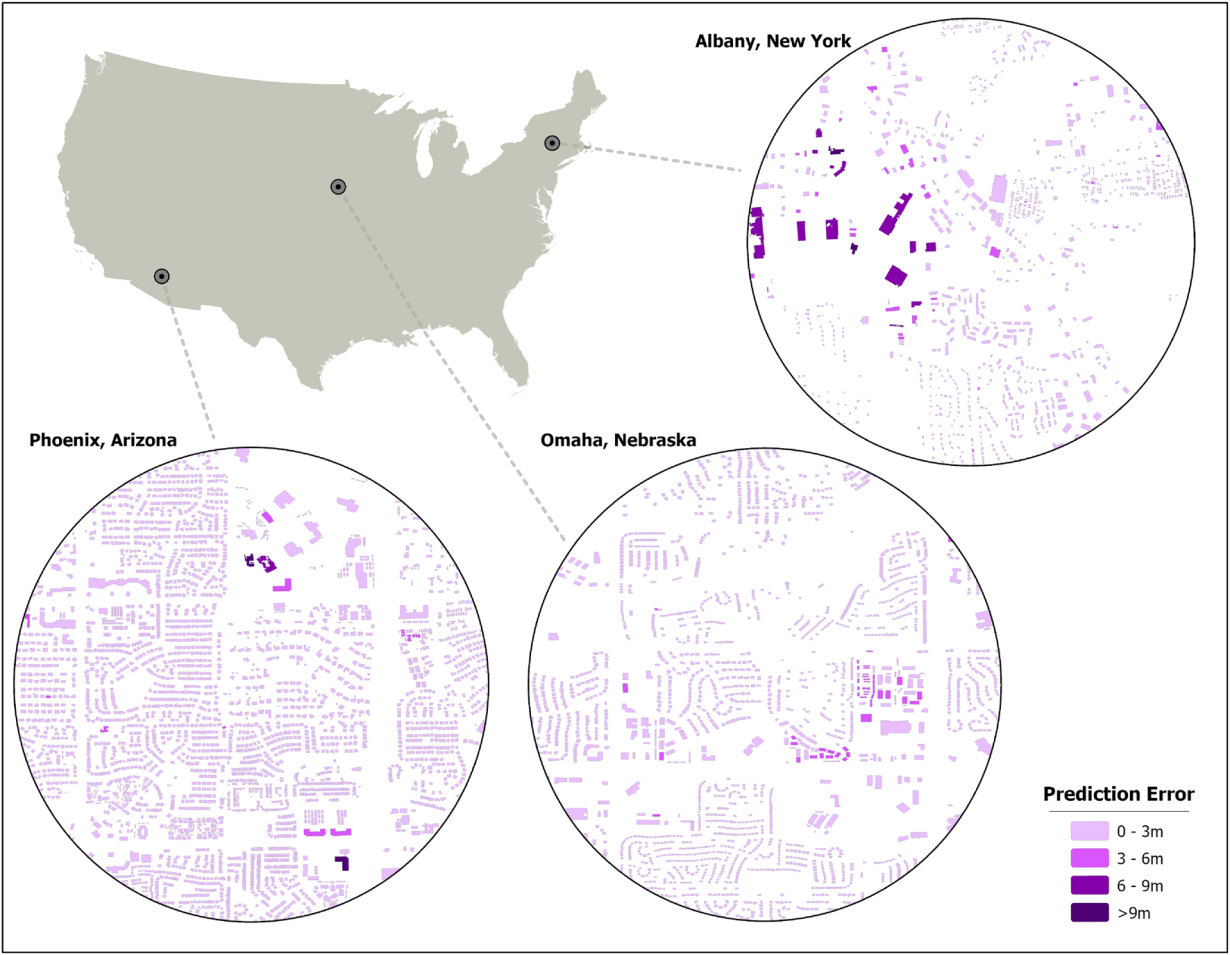
Building footprints with associated prediction error in Phoenix, Arizona, US, Omaha, Nebraska, US, and Albany, New York, US These cities were chosen because high quality validation data were available. Each section shows the error associated with our approach in relation to ground truth height data. While validation data were available for some cities, the majority of buildings within the US (97.78 million) were inferred with no ground truth data available.

In this study, we predict building height using only morphology features derived from individual footprint geometries, distinguishing our approach from previous work that leveraged ancillary data^44,45^. The exclusion of ancillary data makes our approach scalable, thus allowing this method to infer height for an individual building anywhere there is a footprint, regardless of spatial context. This was evidenced as 97% of buildings across 15 out-of-geography cities were predicted within 3 m of their actual height, as demonstrated in experiment I. Furthermore, we do so in a way that is source agnostic, the first time to our knowledge that height has been predicted at the building level through machine learning models that leverage footprint morphologies not derived from lidar imagery. Our results are comparable to the current state-of-the-art machine learning approach with respect to building height accuracy within 2.5 m. Finally, we were able to train the model on selected cities and predict height for 97.78 million buildings in the US, demonstrating the ability of this approach to scale to large geographic areas. We showcase our overall workflow in Fig. 1 and illustrate our prediction accuracy across select cities in the US in Fig. 2.

**Figure 3:**
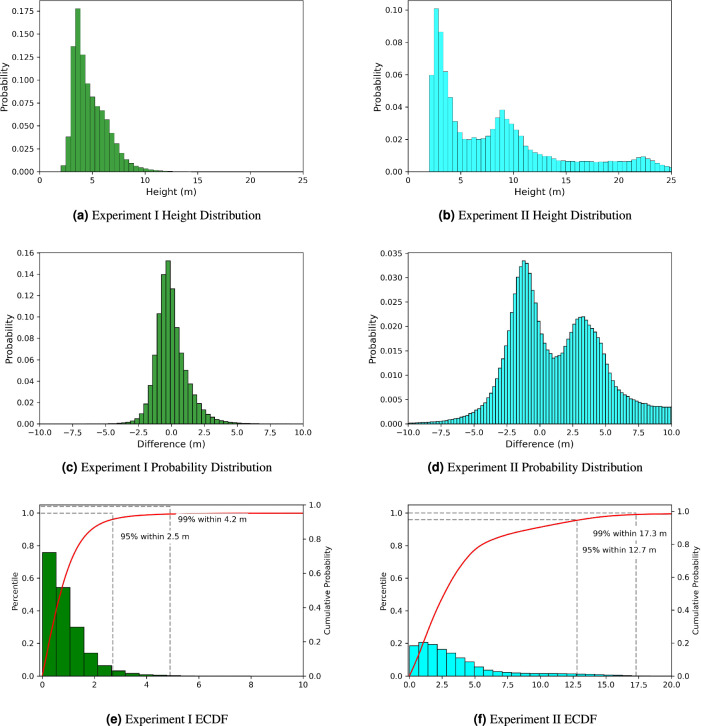
Highlighting the building distribution (top) for experiments I and II. The middle panel displays the error distribution (predicted − actual) for experiments I and II, with the bottom panel showing the empirical cumulative distribution function (ECDF) for each respective experiment. Please note the difference in scale for the ECDF between experiments I ($0{-}10 \; \text{m}$) when compared to II ($0{-}20 \; \text{m}$). All the figures for experiment II are derived from the testing dataset in Berlin, Germany.

## Results

We conducted two experiments on lidar-derived footprints to understand the model’s ability to scale and generalize across geographies and one experiment on satellite-derived footprints to investigate its efficacy on non-lidar footprints. Our first experiment was conducted on buildings within 118 cities across the entirety of the US to test our initial theory of predicting height from 2D building geometries. While we conducted a thorough validation process which involved testing on out-of-sample distribution for experiment I, the samples were within the same country (US) (Fig. S1). The second experiment was done to test the ability to generalize across countries by training in one country (France) and testing in another (Germany). Experiments I and II were based on lidar-derived footprints, which is a constraint on applying our methodology more broadly. Therefore, a third experiment was conducted to address this lidar modality limitation by applying our approach on footprints derived from satellite imagery.

**Table 1:** Results for experiments I–II for MAE, RMSE and $R^{2}$.

Metrics	Experiment I				Experiment II			
	MAE	RMSE	$R^{2}$	CV	MAE	RMSE	$R^{2}$	CV
Median	1.31	2.01	–	–	4.97	6.95	–	–
LR	1.27	1.84	12%	–	4.31	6.26	14%	–
XGBoost	0.91	1.45	45%	1.47	3.82	5.79	26%	–

The metrics in this table represent the results associated with the median, the LR, and XGBoost approach. The top row of the metrics is associated with the median, the simplest possible approach that must be improved upon. The next row, LR, displays the results from the un-tuned linear regression approach with the bottom row, XGBoost, showing the results associated with our tuned approach. For example, for the MAE in experiment I, the median is 1.31 m, which the LR improves upon with a metric of 1.27 m which is further improved with a result of 0.92 m from the tuned XGBoost approach. The cross-validation (CV) score is in relation to the RMSE.

For each experiment, we utilize the gradient boosting trees algorithm, XGBoost, as our favored model and evaluate our results against two different methods for inferring height: the simplest approach, labeled ’Median’, assigns all buildings the median building height from our training data. This quantifies the extent to which any model is an improvement over the simplest possible method for inferring building height. We next apply a Linear Regressor (LR) with default settings; similar to the method of Milojevic-Dupont et al.^45^ and evaluate the mean absolute error (MAE), root mean squared error (RMSE), the standard accuracy method as defined by the American Society for Photogrammetry and Remote Sensing when dealing with non-vegetated terrain, and goodness of fit, $R^{2}$^61^. One of the main differences between our machine learning validations is that LR is a parametric model while XGBoost is a non-parametric model and consistently outperforms LR^62^. Our approach, a tuned gradient boosting trees model builds from^45^ and is labeled XGBoost.

To validate our predictions, we conducted a tenfold cross-validation (CV) during the training of the model to ensure that no over-fitting was occurring for experiments I and III. CV was not done for experiment II as the training data was from France and the testing data was from Berlin, Germany, two separate datasets. However, it has been noted that to ensure generalization for large-scale geographic models, it is necessary to test on spatially independent samples to ensure that nearby training data does not influence the results^63^. Therefore, in addition to the tenfold CV for experiments I and III, we also employed a validation approach similar to that done by Metzger et al.^64^ in which geographically distinct locations are held out for validation as an out-of-sample distribution (Fig. S1, Table S1).

In experiment II, to validate, we compare our approach against current state-of-the-art results from the same testing dataset in Berlin, Germany^45^. It must be noted that our training set for experiment II was derived from buildings in France while in Milojevic-Dupont et al.^45^, the training set was derived from buildings in the Netherlands. France was chosen over the Netherlands as it had a more diverse representation of the built environment.

In experiment III we evaluate model accuracy when the model is run on footprints derived from lidar and footprints derived from satellite imagery. As a further step, we also investigate the difference of our predicted height from footprints derived from satellite imagery across three US cities with Microsoft building heights (https://www.microsoft.com/en-us/maps/bing-maps/building-footprints), a publicly available open-source building height dataset. The three cities were selected based on the availability of Microsoft footprints, satellite-derived footprints, and lidar-derived ground truth heights in an overlapping area. While controlling for temporal variability would be ideal in a best-case scenario, it was difficult to determine the sources and dates for Microsoft footprints and there was variability in the timing of lidar acquisition (Table S2).

**Figure 4:**
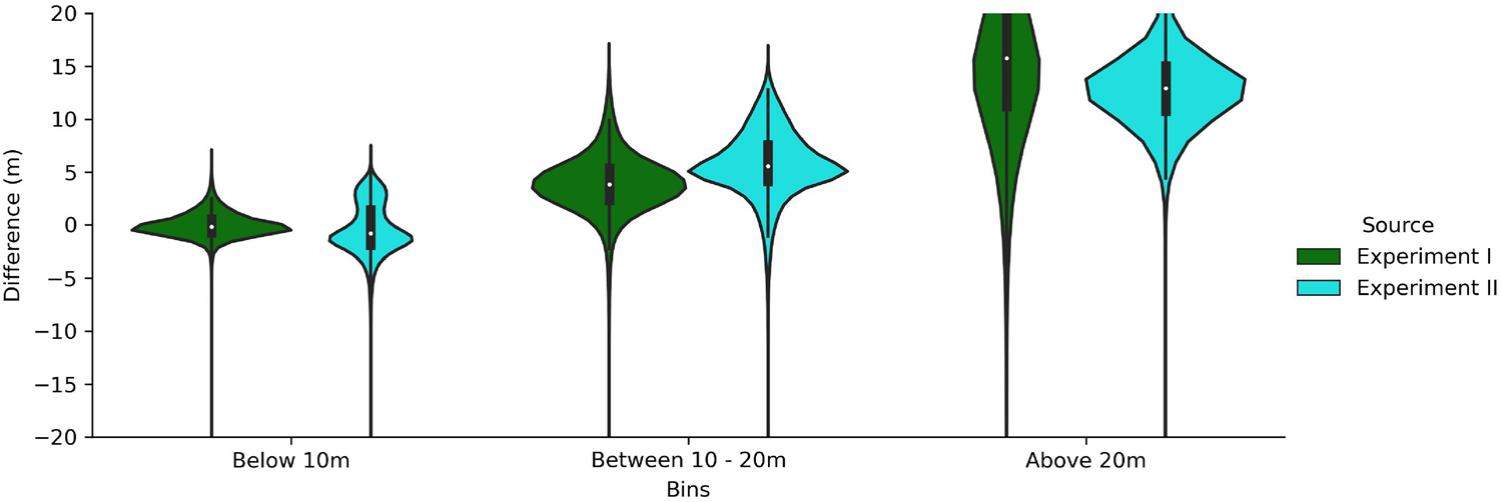
Violin plot for experiments I and II showing the error distribution largely centered around zero for buildings less than 10 m in height. The error increased for buildings greater than 20 m, but the distribution of these buildings were less than 1% in experiment I and 7% for experiment II.

### Experiment I

Our first experiment is composed of 118 cities and 31.41 million buildings in the US (Table S3). Buildings in this experiment have a comparatively low mean height (4.85 m) in relation to experiment II (Table S3). We found that 95% of our predictions from the tuned XGBoost model were within 2.47 m absolute error (predicted height – actual height) and 99% were within 4.24 m (Fig. 3). The high accuracy is partly due to the high percentage (99%) of buildings smaller than 10 m in height within experiment I. The LR showed positive results of 1.27 m for MAE and 1.84 m for RMSE with a $R^{2}$ score of 12% in comparison to the median baselines of 1.31 m MAE and 2.01 m RMSE (Table 1). The tuned XGBoost model improved upon the untrained LR with results of 0.91 m MAE, 1.45 m RMSE and an $R^{2}$ of 45% (Table 1). Each of the 15 cities reserved for the out-of-sample distribution displayed positive $R^{2}$ values, with a low of 3% for Boise, Idaho, US and a high of 40% for Orlando, Florida, US (Table S1, Fig. S2). We hypothesize that the $R^{2}$ of 3% in Boise, Idaho, US was due to a lack of nearby training data. The low $R^{2}$ also seemed to be more present in cities which displayed a bi-model height distribution, as displayed in Los Angeles, California, U.S. and Salem, Oregon, U.S. Multiple studies have documented the increase in accuracy associated with having local training data, sometimes as little as 2%^45^. Regardless, the model improved upon the RMSE from the median and also displayed a positive $R^{2}$ for each of the holdout cities. Furthermore, 97% of the absolute error associated with all of the hold-out cities was within 3 m.

### Experiment II

The second experiment is composed of 41.17 million buildings in France for training and 0.47 million buildings in Berlin, Germany for testing (Table S3). The mean building height of 8.73 m in Berlin, Germany was higher than the mean height from experiment I (Table S3). The median baselines were also higher, showing values of 4.97 m MAE and 6.95 m RMSE. The untrained LR displayed metrics of 4.31 m MAE, 6.26 m RMSE and 14% $R^{2}$ (Table 1). The tuned XGBoost model showed results of 3.82 m for MAE, RMSE of 5.79 m, and $R^{2}$ of 26%. While a direct comparison is unfair due to the differences in training data, it must be noted that 52% of our results were within 2.5 m, with 62% being observed in Milojevic-Dupont^45^. We show that it is possible to predict building height, even with significant variance in building height distribution between the training and testing sets as the top quartile of buildings in our testing dataset (Berlin) includes buildings taller than 11.13 m, while the top quartile of buildings in our training dataset (France) includes buildings taller than 6.23 m (Table S3). Furthermore, the positive $R^{2}$ validates that our approach is generalizable and can train in one country, and infer heights in another.

For experiments I and II, the tuned XGBoost model performed better when predicting buildings less than 20 m in height (Fig. 4). While buildings greater than 20 m composed less than 1% of the distribution for experiment I and $7\%$ for experiment II, they represent socially, economically, and ecologically important contexts. For example, buildings [2, 10 m] had an absolute error less than 6 m, which signifies 2 floors, for all buildings in experiment I and 99% for experiment II. When buildings [10, 20 m] were taken into account, percentages of predictions with absolute error less than 6 m were $81\%$ and $59\%$ for the respective experiments. For buildings $[>20\; \text{m}]$, the error increased for both experiments. Therefore, while our approach did not predict taller buildings well, these buildings represented less than $1\%$ of the height distribution overall.

**Figure 5:**
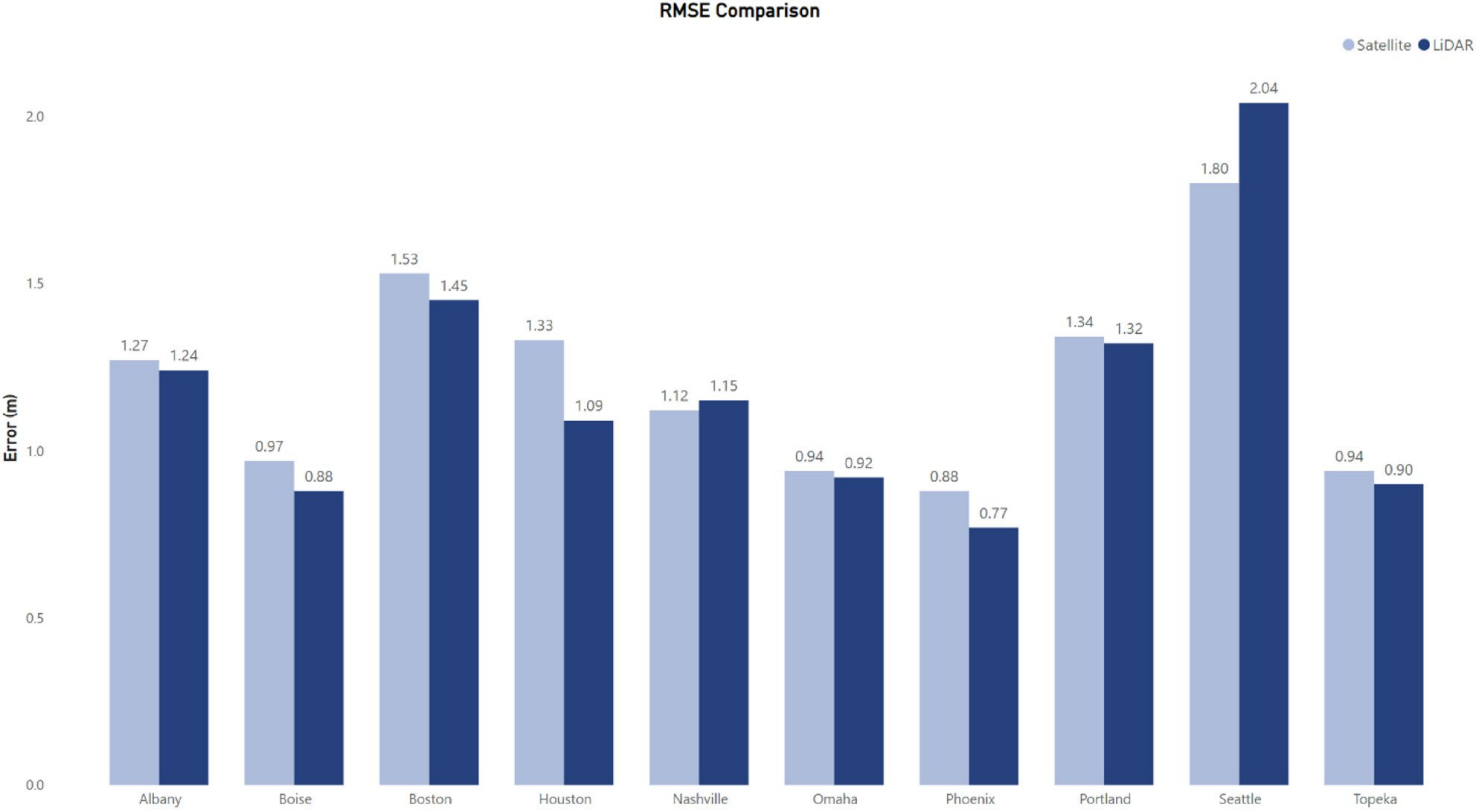
RMSE metrics of the 10 cities in experment III during the XGBoost model run, the highest performing model, for footprints derived from LiDAR imagery (dark blue) and satellite imagery (light blue).

### Explainability analysis

For model explainability, we generated SHapely Additive exPlanations (Shap) values to examine which of our input features displayed significance in relation to predicting the target feature, height in experiments I and II (Fig. S3)^65^. While some features were important across experiments I and II, such as *complexity ratio*, a engineered feature, and the nearest neighbor index (*nni*), a contextual feature, other features varied based on geographic location (Fig. S3). For example, the *ipq*, which represents the shape area maximization for a given perimeter length, another engineered metric, helps identify circular buildings from other shapes, such as a square or rectangle (Fig. S4). This feature was highly influential for experiment I, especially in relation to buildings between 2 and 10 m, but was not influential for any bins in experiment II (Fig. S3). The *complexity ratio*, a feature which can help identify taller buildings based on their unique footprints when compared to smaller buildings in dense urban areas was the third, and second highest feature for the respective experiments in buildings > 20 m (Fig. S3). While engineered values were highly ranked in terms of feature importance, other features such as the number of buildings within a specified distance provide helpful contextual information. In experiment II, *n size max 50*, which calculates the largest building size within 50 m, was in the top five most influential features for each of the bins. The fact that specific features, such as *complexity ratio* and *omd 1000*, consistently show high correlation to height regardless of spatial context and building height is an important finding and one that further solidifies our approach as applicable at scale.

### Experiment III

In experiment III, we predicted height on 1.64 million buildings across 10 U.S. cities where USA structures and lidar coverage overlapped (Table S4). First, we predicted height on these 10 selected cities based on lidar footprints and then ran the model using the same approach based on satellite derived footprints. We found that it is possible to predict building height based on morphology features from footprints derived from satellite imagery as the lowest $R^{2}$ value displayed was $39\%$ in Albany, New York, U.S., with a high value of $55\%$ in Phoenix, Arizona, U.S., both results from a tuned XGBoost model (Table S4). This is comparable to the footprints derived from lidar imagery as the low $R^{2}$ value was $40\%$ in Nashville, Tennessee, U.S. and the high value $58\%$ in Houston, Texas, U.S. (Table S4). While there was a negative value displayed by the LR in Portland, Oregon, U.S. on the satellite derived footprints, the tuned XGBoost model displayed a positive value. The out-of-sample cities all displayed a positive $R^{2}$ value with the first city (Houston, Texas, U.S.) metric of 17%, the second city (Boise, Idaho, U.S.) value of 13%, and the third city (Seattle, Washington, U.S.), a value of 23%. These results for the hold-out cities are similar to experiment I in which all cities displayed a positive $R^{2}$ value and had a lower RMSE when compared to the median baseline. When absolute error was taken into account for the hold-out cities associated with this experiment, 96% of buildings were within 3 m.

When comparing the differences (RMSE Satellite − RMSE lidar) between the cities, there were minor differences with Houston, Texas, U.S. displaying the highest difference (0.24 m) (Fig. 5). On average, the difference in RMSE was 0.04 m between the fooprints derived from satellite imagery with the footprints derived from lidar imagery. Furthermore, in Nashville, Tennessee, U.S. and Seattle, Washington, U.S. the footprints derived from satellite imagery outperformed the footprints derived from lidar imagery by 0.03 m and 0.24 m, respectively (Fig. 5). This shows that our approach of predicting height from footprint morphology information is source agnostic and can be applied when no lidar data is available.

### Microsoft comparison

Of the 10 cities from experiment III, we selected three that had overlapping footprints with Microsoft height values. Microsoft has consistently striven to identify and map building-level features at scale, one of which is building height. Microsoft has developed a Neural Network that infers height on individual building footprints, which undergo a thorough cleaning process, derived from Bing imagery. To validate our approach of inferring height from footprints derived from satellite imagery, we conducted an analytical comparison of our inferred building heights using a XGBoost model with those of Microsoft to identify and characterize the differences. Microsoft building height predictions in each of the three cities we used in the evaluation included -1 values, presumably null values, which we removed during the conflation process.

**Figure 6:**
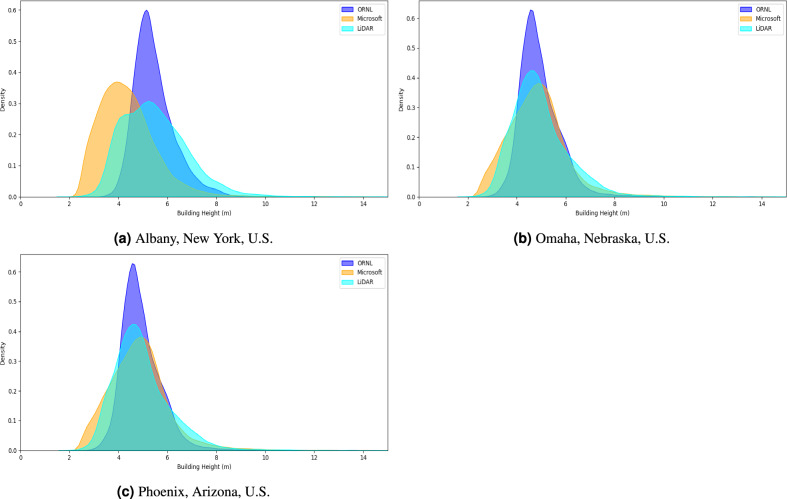
A Kernal Density Estimate displaying the differences exhibited between Microsoft, the approach presented in this paper (ORNL) and lidar for Albany, New York, U.S. (**a**), Omaha, Nebraska, U.S. (**b**), Phoenix, Arizona, U.S. (**c**).

To facilitate a meaningful comparison of the height distributions, we generated kernel density estimation (KDE) plots for both sets of predicted building heights in Albany, New York, U.S., Omaha, Nebraska, U.S., and Phoenix, Arizona, U.S., as seen in Fig. 6. Descriptive statistics for the KDE plots of each city per dataset are shown in Table S5. In the cities of Omaha, Nebraska, U.S. and Phoenix, Arizona, U.S. the central tendencies of our model and that of Microsoft are similar. In Albany, New York, U.S., our model has a higher central tendency, implying that, on average, we tend to estimate larger building heights than Microsoft. Across the three cities evaluated, Microsoft’s predicted building heights show a large standard deviation, indicating that the Microsoft model predicts a broader range of height. Microsoft and our model show positive skewness, indicating that both models are likely to predict a high number of short buildings and a low number of tall buildings (Table S5). However, the Microsoft model shows higher skewness in all three observed cities, implying that it tends to predict a higher number of taller buildings than our model does. Additionally, Microsoft has higher kurtosis in all three cities, reinforcing that their model has a significant presence of large outlier height values.

When evaluating our predictions and Microsoft’s against reference lidar heights, our predictions more closely align with the central tendencies of the lidar heights. Microsoft’s predictions showed greater dispersion and skewness that resembled that of the lidar heights better than our own. In addition to descriptive statistics, error metrics such as MAE and RMSE were also considered to compare the predicted heights of our model and Microsoft’s against lidar measured heights. Our model performed better in each city in relation to MAE and RMSE (Table S5).

These findings suggest that our model not only predicts building heights that are, on average, closer to the lidar measurements but also does so with greater consistency and reliability. Conversely, Microsoft’s predictions exhibit higher variability and a distribution shape similar to the lidar measured building heights, as indicated by the larger standard deviations and higher skewness and kurtosis values. This suggests that while our model provides a more accurate and consistent estimation of building heights, Microsoft’s model captures the broader range and the extreme values in building heights more effectively.

The reason our MAE and RMSE differ from the experiment III’s metrics is due to removing buildings that displayed a − 1 metric in Microsoft’s buildings. For example, $28\%$ of the data in Albany, New York, U.S., totaling 9054 of the 32,455 buildings displayed a − 1 value. This trend was also displayed in the other cities in which we examined, with 5843 of the 48,615 buildings in Omaha, Nebraska, U.S. and 19,336 of the 140,746 buildings in Phoenix, Arizona, U.S. displaying a − 1 value. It must also be noted that we had local training data in these cities, we are unaware if Microsoft had local training data. This is important as local training data is known to improve the model’s performance when using morphology information to infer height as evidenced by our results from experiment I as well as previous research^45,46^.

Therefore, while our approach displayed lower MAE and RMSE, it must be acknowledged that the kurtosis associated with the Microsoft model was higher, thus signifying a higher percentage of tall buildings. It has been noted that predicting taller buildings accurately is a limitation in our approach, as evidenced by a higher RMSE for buildings greater than 20 m in height (Fig. 4). However, our approach displays a lower MAE and RMSE when compared against the lidar derived heights and we are able to predict heights on the footprints which displayed a − 1 value in the Microsoft dataset, thus allowing our approach to encapsulate a larger volume of footprints.

## Discussion

Our proposed approach of inferring height based on 2D footprint information not only shows that it is comparable with previous work which leveraged ancillary data, we do so with favorable results based on footprints derived from satellite imagery when compared to footprints derived from lidar imagery^44,45^. Acquiring lidar data can be expensive and its scope is largely limited to city or state based studies^25,26,28^. Alternatively, there has been a rapid rise in the amount of open-source footprints derived from satellite imagery at a global scale. This signifies that our approach will not be constrained by lidar modality issues, allowing our approach to leverage a more available source, footprints derived from satellite imagery. This expands the ability of our method to map height in areas where current methods were previously unable to, evidenced by mapping height at a building-by-building level for 97.78 million buildings in the U.S. which did not previously have a height metric^44,45^.

One challenge we encountered was the ability for our model to generalize to new countries. The issue of generalization was evident by the different metrics selected by the Bayesian optimization during the model development for each of the three experiments. It is documented that the built environment displays differing characteristics depending on building location and therefore, the approach may need to be tailored based on the geographic location^66^. Regardless, experiment II was able to generalize from buildings in France to buildings in Berlin, Germany with no local training data, evidenced by a $R^{2}$ value of 26%. The ability to train in one country and test in another with no local training data is significant and shows that while different metrics for hyper-parameters may be needed, the approach can generalize. We further showcased the ability for our approach to generalize, evidenced by the positive $R^2$ values associated with each of the out-of-sample cities for experiments I and III.

A structural challenge for building height estimation is the relative rarity of tall buildings compared to short buildings. This class imbalance is a feature of how cities are built around the world as there is a higher frequency of smaller buildings within the built environment in relation to taller buildings. For example, in experiment I, 75% of the building height distribution was within 5.79 m, yet the tallest building was 532.05 m (Table S1). Furthermore, while our model displayed the highest RMSE in relation to buildings greater than 20 m in height, 0.10%, and 9.27% of buildings were greater than 20 m in height for experiments I and II (testing). Regardless, this is a limitation of our approach and further work is needed to accurately map taller buildings. Previous studies have documented possible ways to identify taller buildings and in some cases simply binned buildings into “taller” and “smaller” categories^67–69^. However, there is debate about what quantifies a “taller” building with values ranging from 22.5 m in the U.S. to 70 m in China^70,71^. Additional analysis must be done to assess if it is possible to quantify what defines a taller building at a global level to help researchers compare studies spatially.

We intentionally included all buildings greater than 2 m in the data and did not bin them. While we acknowledge that there may be some areas in the world where inhabitable structures are less than 2 m in height, we wanted our approach to remain consistent with previous studies for better comparison^45^. Furthermore, specific countries have varying definitions of minimum heights for habitable rooms, such as 2.70 m in Italy and 2.60 m in the Netherlands and Portugal while there are no limits in England or Wales^72^. Thus, rather than changing the minimum height at a country-by-country level, we applied a consistent method that allows us to compare to previous work^45^.

In addition to the high error related to taller buildings, another limitation of our work is the differences in footprint shapes between lidar footprints in relation to satellite footprints. For example, if there is overhanging vegetation or cloud coverage, this will affect the footprint shape of the building of interest for one data, especially when there is a temporal gap in relation to when the footprints were collected, as is our case (Table S2). This can lead to a footprint that is not representative of the overall shape of the corresponding building within the image where height is being inferred. Another issue can often be seen in cities and dense urban areas in which one large polygon can represent multiple different structures, such as strip malls, row houses, and buildings with walkways that connect multiple buildings (Fig. S5). Furthermore, false positives can also effect the outcome as there are footprints that may show up as buildings in one data and not the other, such as tennis courts (Fig. S6). In this scenario, some of the contextual features may show slight differences when shorter distances (50 m, 100 m) are taken into account (Table 2). Regardless, we employed a strict conflation process to ensure that there were minimal instances when false positives would effect our model, as evidenced by our overall absolute error being within 3 m for 95% of the buildings.

We demonstrate that it is possible to predict building height at a building-by-building level across large geographic areas using machine learning based only on morphology features derived from building footprints. Our model results across 74.55 million buildings show that 95% of the error was within 3 m. We base our approach on one widely available input source, building footprints, and do not require ancillary data, such as roads. Furthermore, we showcase our novel ability to infer height based on footprints derived from satellite imagery, the first time to our knowledge this has been accomplished at a building-by-building level based on footprint morphology features. Further work is needed to fully understand the built environment, especially in regard to taller buildings, a limitation of our application. Nevertheless, this work represents an advancement in relation to mapping the built environment and is a foundational building block for future studies. The implication of this new approach to inferring height from footprint morphology data alone has applications across a broad swath of domains. Previous studies have pointed out the importance of building height in relation to population studies, which can help improve understanding of natural disaster operations, epidemiological modeling, identifying populations at risk of climate and land change, urban energy use and infectious diseases and hazards^9,10,73–76^. UHI’s in particular are of high importance and building height is a key metric needed to further the understanding of how UHI’s can affect public health^8,11,12^. We provide researchers with a method to help quantify and understand expanding cities across a broad swath of applications at a global scale.

## Methods

### Data

For our analysis in experiments I–III, we leverage 74.55 million building records across the U.S., Germany, and France. These regions have different building ages, morphologies, and height distributions and therefore provides a natural experiment to evaluate model sensitivity to those characteristics^22^. The data used in this study are all open-source and available through publicly accessible websites. The individual country datasets are described in more detail below.

#### U.S. buildings

Building footprints and height information for the United States were provided by the USA Structures building inventory (https://gis-fema.hub.arcgis.com/pages/usa-structures). This dataset is a compilation of footprints derived from satellite imagery and lidar derived building footprints with associated attributes and was developed by Oak Ridge National Laboratory (ORNL), Federal Emergency Management Agency Geospatial Response Office, and the Department of Homeland Security Science and Technology Directorate^77^. Over 100 cities in the U.S. contain lidar derived building footprints which were acquired by the National Geospatial-Intelligence Agency between 2003 and 2015 and declassified in 2017. The height associated with these lidar derived footprints is the average of the height above ground values for all rooftop lidar points associated with a structure. The datasets for individual cities used in this study are part of that subset of data.

**Table 2:** Building morphology features.

Feature	Description
**Shape area**	Area of polygon in un-projected units
Shape length	Perimeter length in un-projected units
sqft	Area in square feet
lat dif	Maximum latitude minus minimum latitude in un-projected units
**Long dif**	Maximum longitude minus minimum longitude in un-projected unis
Envel area	Area of bounding box of geometry in un-projected units
**Vertex count**	Count of vertices in geometry
geom count	Count of polygons in the geometry
**Complexity ratio**	Shape length/shape area
**iasl**	Inverse average segment length
**vpa**	Vertices per area
**Complexity ps**	Complexity per segment, average complexity within each segment
**ipq**	Isoperimetric quotient, shape area maximization for given perimeter length
sqmeters	Area in square meters
**n count**^a^	Number of building centroids within a given distance
**omd**^a^	Observed mean distance from building within a given distance
emd^a^	Expected mean distance from building within a given distance
**nnd**	Nearest neighbor distance from building
**nni**^a^	Nearest neighbor index, overall pattern of points within a given distance
Intensity^a^	Amount of nni occurring
**n size mean**^a^	Average size of buildings within a given distance
**n size std**^a^	Standard deviation of buildings within a given distance
**n size min**^a^	Smallest building size within a given distance
**n size max**^a^	Largest building size within a given distance
**n size cv**^a^	Coefficient of variation of building size within a given distance

^a^Denotes feature being calculated on multiple scales. Bolded features highlight the features selected for use

#### German buildings

Building data for Berlin, Germany is provided by the Business Location Center (https://www.businesslocationcenter.de/en/economic-atlas/download-portal/). The dataset includes a 3D mesh model based on airborne lidar through a collaborative effort by the Senate Department for Economic Affairs, Energy and Public Enterprises, and Open Data Initiative of the state of Berlin. To process the GML files provided we followed the steps presented in^45^.

#### French buildings

Building data for France is provided by Eubucco (https://eubucco.com/data/)^78^. Eubucco is an open-source dataset and is composed of over 50 datasets that provide information on building characteristics and height for 27 European countries and Switzerland. For our purpose we leveraged the country of France from this dataset.

#### Microsoft buildings

Microsoft provides building data for multiple continents (https://www.microsoft.com/en-us/maps/bing-maps/building-footprints). We leveraged their coverage of 3 diverse cities within the U.S. where there were overlapping footprint data with lidar and USA Structures satellite derived footprints.

### Morphology features

The Geographic Augmentation of Extracted Buildings Features Tool (Gauntlet) generates morphology features from vector geospatial polygon data layers^50^. The morphology feature set consists of three types of features: geometric, engineered, and contextual. Geometric are basic measures of geometry like area or perimeter. Engineered features describe more complex ideas like compactness or complexity. Contextual features describe the building and its relationship to its neighbors, both spatially and in size. The contextual features are generated at five different scales: 50 m, 100 m, 250 m, 500 m, and 1000 m. Overall, there are 65 features generated with table 2 providing a description of each feature. The Gauntlet feature set was generated for all building datasets discussed in data.

### Feature selection

All buildings under 2 m in height were removed from each dataset and the remaining feature data were then standardized. Feature reduction, which can improve model efficiency, was conducted in order to reduce dimensionality, and as a result, decrease model complexity^79^. We leveraged recursive feature elimination (RFE) with a XGBoost regressor with default settings from the xgboost 1.7.5 library in python^80^. This technique iteratively removes the least influential feature until the features which show the highest impact on predicting height remain. This process was done on a compiled dataset which consisted of all the lidar footprints, as well as a dataset which consisted of all the satellite footprints. The RFE process resulted in narrowing our list of morphology features listed in Table 2 to the following 30, for the lidar footprints in experiments I-III (highlighted in bold in Table 2): *nnd*, *shape area*, *long dif*, *vertex count*, *complexity ratio*, *iasl*, *vpa*, *complexity ps*, *ipq*, *omd 50, 100, 250, 500, 1000*, *n size max 50, 100*, *n size min 100, 250, 500, 1000*, *nni 250, 500, 1000*, *n size std 250, 1000*, *n size cv 250, 1000*, *n count 500*, *n size mean 500, 1000*. The following features were selected for by the RFE for the satellite footprints in experiment III: *shape area*, *long dif*, *envel area* *complexity ratio*, *n count 50, 1000*, *n size max 50, 100, 1000*, *omd 100, 500, 1000*, *n size mean 100, 250, 500, 1000*, *n size min 100, 250, 500*, *nni 250, 500, 1000*, *n size std 500*.

### Experimental design

We conducted two experiments on lidar footprints alone to understand the model’s ability to scale and generalize across geographies. Experiment III was conducted to test the ability of our approach to transfer from footprints derived from lidar imagery to footprints derived from satellite imagery.

#### Experiment I

Experiment I was conducted on 118 cities in the U.S. and included 31.41 million buildings (Table S3). This experiment was done to test our initial theory of predicting height from morphologies derived from individual building footprints.

#### Experiment II

Experiment II was conducted in Europe and involved training the model in France (41.17 million buildings) and testing in Berlin, Germany (0.47 million buildings) (Table S3). This analysis was similar to that done by Milojevic-Dupot et al.^45^ and was carried out to validate the results against the current state-of-the-art. In addition, we also wanted to test the approach across countries without including local data during model training.

#### Experiment III

In experiment III we seek to understand if our methods will transfer to building footprints derived from satellite imagery. USA Structures^81^ provides building footprints extracted from lidar and satellite imagery. These geometries are differentiated by the SOURCE field in the schema of USA Structures. Records with a SOURCE domain value of ORNL are satellite derived building detections. We selected 10 cities across the U.S. which included 1.64 million buildings (Table S4).

To acquire building heights derived from lidar imagery for footprints derived from satellite imagery we conducted a thorough conflation process. However, before the conflation can be performed, pre-processing steps need to be completed in the following order: (1) Identify satellite derived footprints within the same coverage of the lidar generated footprints, (2) regularize the satellite derived footprints using the same method as the rest of the buildings in USA Structures, (3) generate gauntlet features for new satellite footprints, (4) spatially join new regularized footprints to their lidar counter parts, and (5) transfer the height value of the lidar ground truth polygon to the regularized building footprint. We then transferred the height from the lidar footprints to the satellite footprints after undergoing a strict conflation process to ensure that the structures were the same building (Table S6).

Conflation of two different building data sets from two different sources that are temporally different is a significant challenge. We impose a strict one to one relationship requirement for this experiment. If a lidar generated footprint overlaps with more than one satellite footprint, all geometries are excluded from the experiment (Fig. S5). This is true for the inverse, if a satellite footprint overlaps with more than one lidar footprint all geometries are excluded. Additionally, there are certain instances when then the conflated footprints differ vastly in relation to shape area (Fig. S5). To compare with the Microsoft footprints we followed the same conflation rules above. It is important to emphasize that this approach has resulted in a non-representative sample of the overall building population within the cities reviewed. Consequently, the resulting descriptive statistics of the distributions observed in our comparative analysis reflect the distribution within this specific sample rather than the entire spectrum of building heights in the real world. Given this context, direct extrapolation of our findings to suggest that one model more accurately reflects real-world distributions would be inappropriate and potentially misleading.

### Model development

XGBoost is a supervised machine learning algorithm that uses a gradient boosting framework to solve classification and regression problems. The XGBoost algorithm, first published by Chen and Guestrin in 2016, builds upon the RF algorithm and operates iteratively by including new trees^82^. One of the main differences between gradient boosting and a tree based model is that each new tree works to correct the error from the previous tree^82,83^. XGBoost is a widely used algorithm and has been applied to research in fraud detection, landslide susceptibility, forecast rice production, bankruptcy prediction, building use type, among others^55,84–87^.

For the model training in experiment I we deployed a 70/30 split from the 118 selected cities, with 70% of the data used for training and 30% for testing. For experiment II, we trained on the buildings in France and applied the model to buildings in Germany. In experiment III, we did a 70/30 split for each of the 10 cities selected for buildings derived from both lidar and satellite imagery.

To fine-tune the XGBoost regressor, we deployed Bayesian optimization via the Hyperopt library^88^. Bayesian optimization works by leveraging the performance from a prior set of hyper-parameters to advise the selection of the successive set of hyper-parameters to test. Once it completes this thorough process, it selects the optimum hyper-parameters from a pre-defined space range as defined by the lowest RMSE. The following hyper-parameters were fine-tuned: *number of estimators*, *max depth*, *gamma*, *reg alpha*, *reg lambda*, *colsample bytree*, *min child weight*, and *learning rate*. In Table S7 we display the optimum hyper-parameters selected for experiments I-III.

### Validation

We conducted a tenfold CV during the training of the model and compared it with the 30% testing set to ensure that no over-fitting was occurring for experiments I and III (Tables 1, S4). However, it has been noted that CV alone is not adequate when working with spatially diverse data and validation should be evaluated on data from distinct geographic regions^89^. To address for this, we conducted a spatial validation similar to that done by Metzger et al.^64^ in which the model is trained in one geographic area, and tested in another. We selected 15 cities across the U.S. to holdout from the training data in experiment I which represent a diverse sample of the built environment^22^ (Fig. S1). For experiment III, we randomly selected 3 of the 10 cities and trained on the remaining 7 cities and then tested on the 3 hold-out cities.

### Explainability analysis

To provide explainability of the model, we leveraged Shap analysis to assess the level of importance features have in predicting building height and providing explainability and transparency to model behavior (Fig. S3)^65,90^. Shap values, which originated from game theory, are contribution scores and provide a consistent and interpretable framework to investigate the features which display high or low influence on inferring the target feature, height^65,90^. We generated Shap values for the features selected for during our RFE for experiments I and II in relation to the three height bins we analyzed: [$2{-}10\; \text{m}$], [$10{-}20\; \text{m}$], and [$>20 \; \text{m}$].

### Application

To showcase the ability of our approach to be applied to other geographic regions without any local data, we trained on the satellite footprints from the 10 cities selected in experiment III and inferred a height value on the remaining satellite footprints, 97.78 million, from the USA Structures dataset that did not have a height attribute. While we did not have any local training data associated with the remaining 97.78 million buildings within the U.S., our validation approach proved that while having local training can improve model results, it is not a requirement.

### Supplementary Information

Supplementary Information.

## Data Availability

The footprint data is publicly available at the URL links provided. To obtain the building heights, the code necessary to run the workflow is available upon request from the corresponding author.
