# Supplementary material for: Inferring building height from footprint morphology data
Source: Sci Rep. 2024 Aug 12;14:18651. doi: 10.1038/s41598-024-66467-2 (PMC11319631; doi:10.1038/s41598-024-66467-2)
Supplement: Supplementary file 1 — Supplementary Information. [file 41598_2024_66467_MOESM1_ESM.pdf]

Supplementary Data

Clinton Stipek, Taylor Hauser, Daniel Adams, Justin Epting, Christa Brelsford, Jessica Moehl, Philipe Dias, Jesse Piburn, Robert Stewart

In our supplementary data section, we have six figures and seven tables:

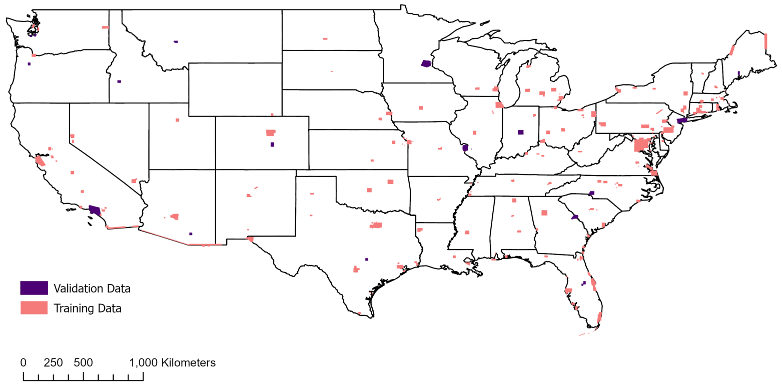

Fig. S1: This map displays the extent of experiment I. There are 118 cities used (pink) for the initial analysis of predicting height from 2D features alone, with the hold-out cities (purple) validating our approach. These 15 hold-out cities represent out-of-sample distribution and are geographically distinct from the training data.

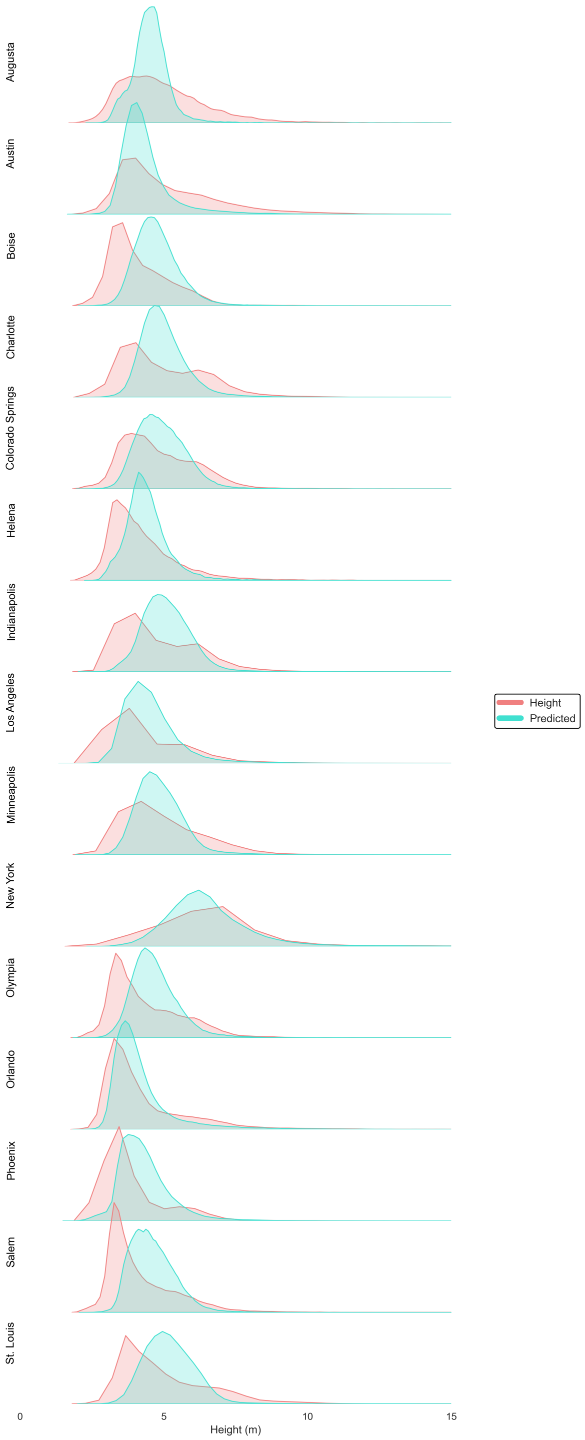

Fig. S2: Ridgeline plots for the height distribution associated with each of the hold-out cities, ordered alphabetically. The predicted height is overlaid the height distribution.

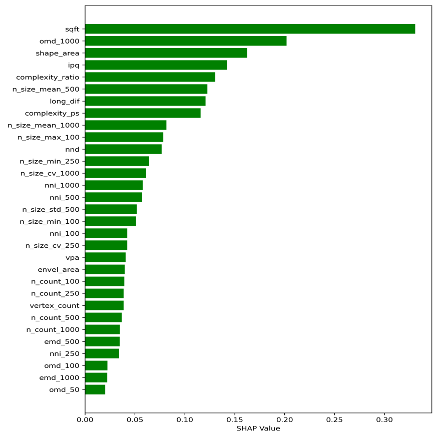

(a) Experiment I $2-10m$

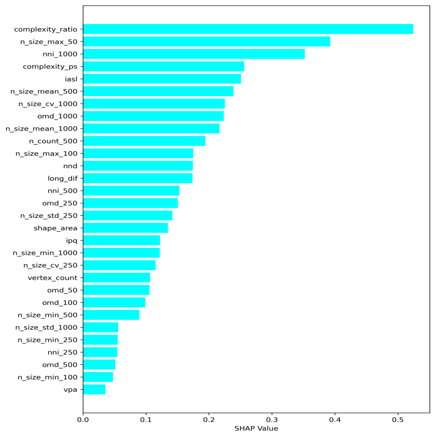

(b) Experiment II $2-10m$

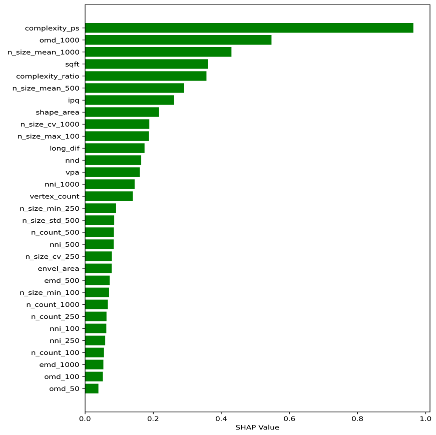

(c) Experiment I $10-20m$

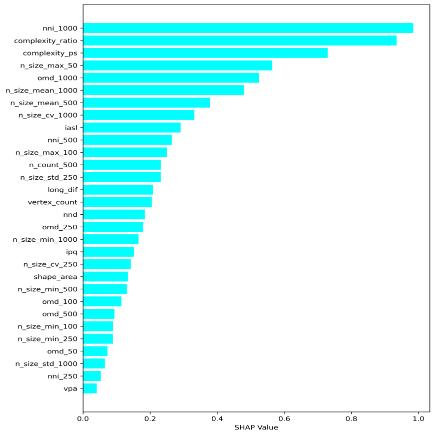

(d) Experiment II $10-20m$

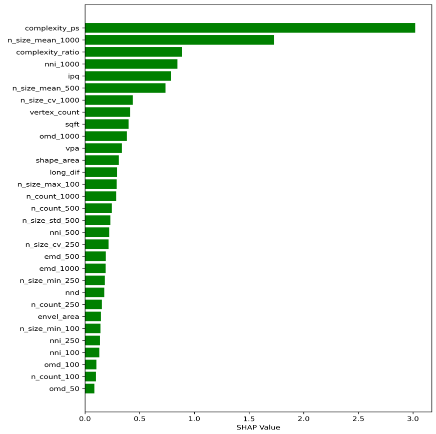

(e) Experiment I $>20m$

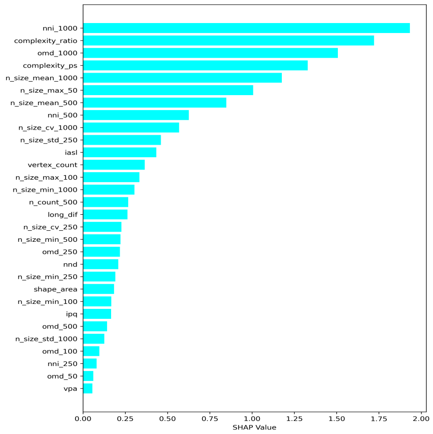

(f) Experiment II $>20m$

Fig. S3: SHAP features for experiments I and II for buildings between $[2-10m]$ on the top row, $[10-20m]$ middle row, and $[>20m]$ on the bottom row.

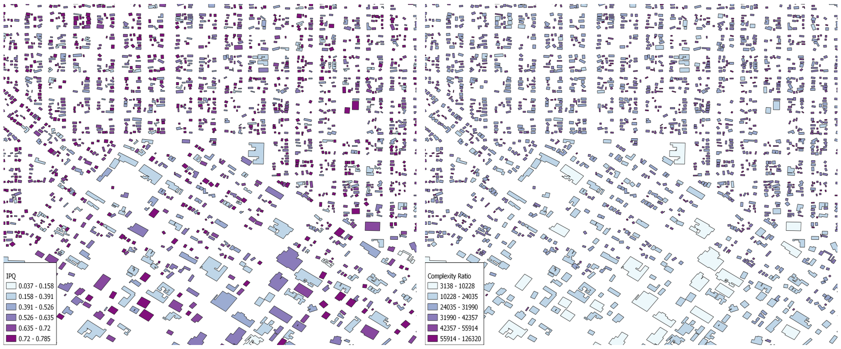

Fig. S4: Here we show two gauntlet features that displayed high significance in relation to predicting height. On the left, IPQ is shown and the complexity ratio is shown on the right, with higher values signifying a more complex footprint.

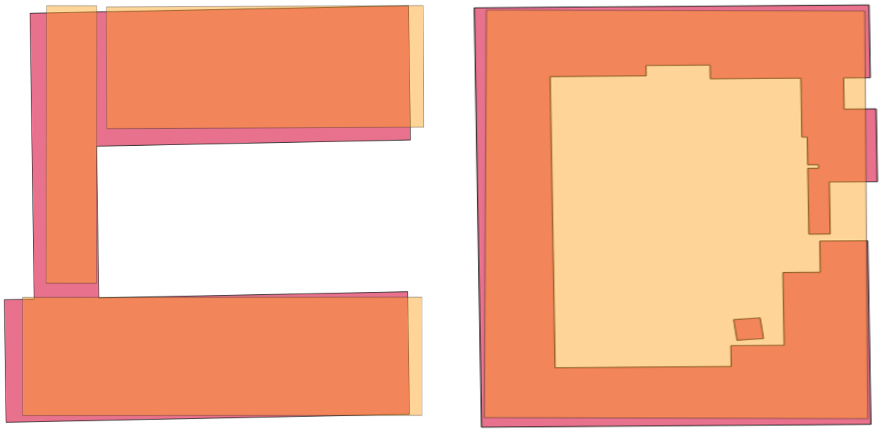

Fig. S5: Here we highlight the issues associated with conflating footprints from different sources. On the left we show a multiple to one scenario in which there are 3 footprints derived from a lidar (orange) source overlaid on one footprint derived from satellite imagery (pink). An example of when there is a large area difference between the two sources is displayed on the right.

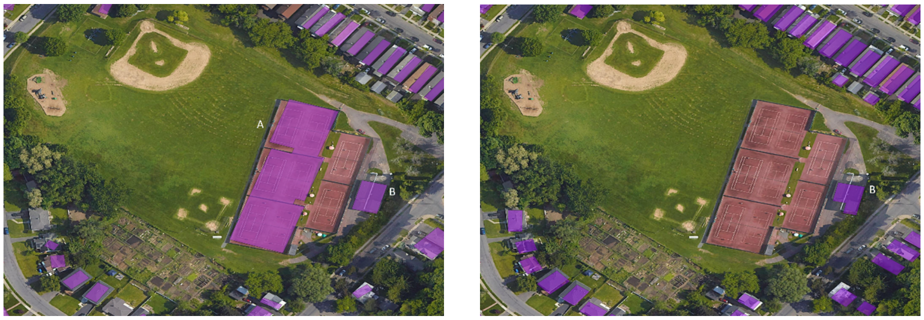

(a) False Positive - Satellite

(b) False Positive Removed - lidar

Fig. S6: This figure shows an example of a false positive. On the left (a) is satellite derived structures. On the right (b) is lidar derived structures. The large polygon that covers a portion of the tennis court is a false positive labelled polygon A. The smaller polygon to the right of polygon A is a true positive and exists in both datasets, polygon B. For polygon B, it was predicted at $5.96m$ when derived from the satellite imagery and $5.62m$ when derived from lidar imagery with the ground truth height being $4.83m$.

TABLE S1: Metrics for experiment I hold-out cities.

	**Augusta**	**Austin**	**Boise**	**Charlotte**	**Colorado Springs**	**Helena**	**Indianapolis**	**Los Angeles**	**Minneapolis**	**New York**	**Olympia**	**Orlando**	**Phoenix**	**Salem**	**St. Louis**
Sample Size	17,125	86,104	126,154	219,792	174,334	26,379	382,852	2,506,351	738,532	552,577	72,060	171,980	779,402	83,221	468,633
MAE - Baseline	1.13 m	1.41 m	0.85 m	1.24 m	1.03 m	0.87 m	1.12 m	0.97 m	1.10 m	1.77 m	1.03 m	1.06 m	0.83 m	0.89 m	1.21 m
MAE - XGBoost	1.02 m	1.28 m	0.96 m	1.05 m	0.83 m	0.81 m	0.97 m	1.02 m	0.91 m	1.58 m	0.92 m	0.83 m	0.85 m	0.93 m	1.11 m
RMSE - Baseline	1.58 m	2.39 m	1.29 m	1.85 m	1.38 m	1.31 m	1.62 m	1.77 m	1.67 m	4.18 m	1.54 m	1.94 m	1.40 m	1.35 m	1.85 m
RMSE - XGBoost	1.46 m	2.10 m	1.24 m	1.53 m	1.12 m	1.13 m	1.34 m	1.57 m	1.36 m	3.48 m	1.26 m	1.43 m	1.23 m	1.21 m	1.57 m
R^2	12%	18%	3%	28%	31%	21%	27%	12%	31%	30%	28%	40%	17%	11%	22%

This table displays the count for each of the respective hold-out cities for experiment I as well as the MAE and RMSE for the median, and XGBoost for comparison. The MAE for the XGBoost was lower in the instances of Boise, Idaho, U.S., Los Angeles, California, U.S., Phoenix, Arizona, U.S., and Salem, Oregon, U.S. However, the RMSE improved upon the baseline and the R^2 was also positive.

TABLE S2: Temporality of lidar, USA Structures, and Microsoft.

**Location**	**Lidar**	**USA Structures**	**Microsoft**
Albany, NY, USA	10/9/2012	19/10/2019	26/4/2023
Boise, ID, USA	8/3/2013	4/8/2018	26/4/2023
Boston, MA, USA	20/5/2009	9/11/2019	26/4/2023
Houston, TX, USA	22/1/2010	21/10/2021	26/4/2023
Nashville, TN, USA	6/6/2006	6/6/2019	26/4/2023
Omaha, NE, USA	24/4/2013	20/5/2020	26/4/2023
Phoenix, AZ, USA	4/10/2014	27/2/2020	26/4/2023
Portland, OR, USA	20/9/2010	27/2/2020	26/4/2023
Seattle, WA, USA	6/5/2010	3/3/2020	26/4/2023
Topeka, KS, USA	10/12/2008	23/11/2020	26/4/2023

The dates for the lidar, USA Structures and Microsoft data listed in DD/MM/YYYY format. It is important to distinguish between the definitions for each date, as the LiDAR date is when the data was collected and the Microsoft date is simply the release date of their product.

TABLE S3: Building Summary Statistics for experiments I-III

	**Experiments**			
**Metrics**	***I***	***II - Test***	***II - Train***	***III***
Count (million)	31.41	0.47	41.17	1.64
Mean	4.85 m	8.73 m	5.24 m	4.71 m
Median	4.38 m	7.09 m	4.71 m	4.19 m
Std	2.00 m	6.75 m	2.67 m	1.82 m
Min	2.01 m	2.01 m	2.10 m	2.01 m
25%	3.56 m	3.36 m	3.62 m	3.45 m
75%	5.79 m	11.13 m	6.23 m	5.57 m
Max	532.05 m	158.81 m	286.32 m	180.90 m

The metrics in this table represent the building height associated with experiments I-III. For example, in experiment I, 25% of the buildings are within $3.56m$ in height and 75% within $5.79m$.

TABLE S4: Experiment III metrics.

**Metric**	**Albany**	**Boise**	**Boston**	**Houston**	**Nashville**	**Omaha**	**Phoenix**	**Portland**	**Seattle**	**Topeka**
Count	116,518	74,901	198,631	261,225	59,597	181,672	522,666	122,048	52,219	56,038
Median MAE	1.15 m	0.85 m	1.48 m	0.96 m	0.90 m	0.91 m	0.79 m	1.16 m	1.33 m	0.82 m
Median RMSE	1.63 m	1.30 m	2.02 m	1.90 m	1.55 m	1.35 m	1.33 m	1.77 m	2.49 m	1.31 m
	**Lidar**									
LR MAE	1.10 m	0.75 m	1.27 m	0.90 m	0.83 m	0.85 m	0.70 m m	1.12 m	1.21 m	0.78 m
LR RMSE	1.51 m	1.07 m	1.75 m	1.37 m	1.30 m	1.20 m	1.07 m m	20.4 m	2.34 m	1.14 m
LR R^2	13%	26%	23%	34%	24%	18%	29%	-31%	23%	20%
XGBoost MAE	0.84 m	0.57 m	1.01 m	0.59 m	0.67 m	0.60 m	0.45 m	0.84 m	0.99 m	0.56 m
XGBoost RMSE	1.24 m	0.88 m	1.45 m	1.09 m	1.15 m	0.92 m	0.77 m	1.32 m	2.04 m	0.90 m
XGBoost R^2	42%	50%	47%	58%	40%	52%	**65**%	45%	41%	51%
CV	1.21 m	0.91 m	1.51 m	1.28 m	1.17 m	0.95 m	0.79 m	1.25 m	1.76 m	0.95 m
	**Satellite**									
LR MAE	1.10 m	0.77 m	1.25 m	0.97 m	0.86 m	0.84 m	0.71	1.13 m m	1.23 m	0.78 m
LR RMSE	1.53 m	1.15 m	1.75 m	1.64 m	1.30 m	1.19 m	1.13 m	1.61 m	1.97 m	1.12 m
LR R^2	12%	23%	25%	19%	19%	19%	26%	15%	28%	18%
XGBoost MAE	0.85 m	0.61 m	1.03 m	0.68 m	0.72 m	0.62 m	0.48 m	0.87 m	1.03 m	0.59 m
XGBoost RMSE	1.27 m	0.97 m	1.53 m	1.33 m	1.12 m	0.88 m	1.33 m	1.34 m	1.80 m	0.94 m
XGBoost R^2	39%	46%	43%	47%	42%	49%	**55**%	41%	40%	42%
CV	1.26 m	0.91 m	1.50 m	1.34 m	1.24 m	0.97 m	0.81 m	1.31 m	1.92	1.01 m

Please note that the top panel are model results from the lidar footprints with the model results derived from satellite imagery on the bottom panel. For the metrics, the LR results are listed above the XGBoost results for both the lidar and Satellite imagery. The CV scores are in relation to RMSE.

TABLE S5: Descriptive Statistics, MAE, and RMSE of Building Heights in Microsoft Comparison study.

City	Model	Mean	Median	Std Dev	Skewness	Kurtosis	MAE	RMSE
Albany, NY, USA	ORNL	5.47 m	5.30 m	0.88 m	1.49	5.56	0.80 m	1.15 m
	Microsoft	4.37 m	4.21 m	1.21 m	1.87	10.49	1.36 m	1.72 m
	Lidar	5.52 m	5.36 m	1.44 m	2.54	29.54	-	-
Omaha, NE, USA	ORNL	4.94 m	4.79 m	0.84 m	1.92	13.30	0.60 m	0.88 m
	Microsoft	4.84 m	4.78 m	1.29 m	2.50	26.40	0.99 m	1.40 m
	Lidar	4.96 m	4.77 m	1.24 m	2.72	28.21	-	-
Phoenix, AZ, USA	ORNL	3.91 m	3.61 m	0.95 m	2.44	15.05	0.47 m	0.78 m
	Microsoft	4.16 m	3.97 m	1.02 m	2.73	26.74	0.67 m	0.93 m
	Lidar	3.93 m	3.53 m	1.23 m	3.63	45.02	-	-

TABLE S6: Building Count by City.

Location	Satellite Footprints	Conflated Footprints	Percent Matched
Albany, NY, USA	143,443	116,518	81%
Boise, ID, USA	116,506	74,901	48%
Boston, MA, USA	256,235	198,631	78%
Houston, TX, USA	503,617	261,225	52%
Nashville, TN, USA	96,816	59,597	62%
Omaha, NE, USA	275,958	181,672	66%
Phoenix, AZ, USA	787,075	522,666	66%
Portland, OR, USA	253,142	122,048	48%
Seattle, WA, USA	126,468	51,219	40%
Topeka, KS, USA	69,749	56,038	80%
Total	2,628,379	1,644,515	63%

The location and the satellite derived footprints with the number that were conflated to the lidar derived footprints to enable us to attach the height associated with each building. To ensure the highest level of integrity during this process we ensured a strict 1:1 conflation process. Overall, of the 2.62 million satellite footprints, we were able to transfer height to 1.64 million footprints across the 10 cities selected for experiment III.

TABLE S7: Hyper-parameters selected for experiments I-III.

	**Albany**	**Boise**	**Boston**	**Houston**	**Nashville**	**Omaha**	**Phoenix**	**Portland**	**Seattle**	**Topeka**	**Exp. I**	**Exp. II**
**Hyper-parameters**	**Lidar**											
Estimators	195	179	192	173	143	191	167	114	116	99	480	345
Max Depth	13	18	18	19	20	10	15	20	14	18	14	14
Gamma	2.99	1.13	0.68	0.89	0.47	0.01	0.04	5.57	0.58	2.42	1.01	6.13
Reg Alpha	2.93	4.39	0.94	3.81	4.01	4.43	7.92	7.41	4.95	4.59	34	60
Reg Lambda	5.55	0.87	1.42	4.01	5.44	1.30	0.78	2.60	5.49	2.33	0.09	0.72
Colsample Bytree	0.62	0.56	0.69	0.69	0.84	0.69	0.54	0.52	0.73	0.58	0.86	0.85
Min Child Weight	3	2	9	3	3	8	6	10	8	9	6	13
Learning Rate	0.10	0.06	0.28	0.04	0.11	0.19	0.14	0.10	0.11	0.12	0.15	0.17
	**Satellite**											
Estimators	199	180	149	111	156	164	161	151	155	126	-	-
Max Depth	18	19	20	20	15	19	17	20	18	16	-	-
Gamma	0.04	0.96	1.43	0.12	2.49	0.91	0.03	6.54	7.51	0.05	-	-
Reg Alpha	6.42	0.87	7.73	9.03	7.90	3.83	8.43	2.16	6.97	3.62	-	-
Reg Lambda	0.95	5.78	4.50	3.95	3.60	3.51	6.11	2.75	0.40	1.91	-	-
Colsample Bytree	0.93	0.61	0.97	0.71	0.75	0.74	0.99	0.79	0.60	0.82	-	-
Min Child Weight	10	4	5	9	6	9	5	8	10	8	-	-
Learning Rate	0.07	0.04	0.07	0.07	0.09	0.04	0.19	0.08	0.03	0.07	-	-

Please note that the top panel of hyper-parameters were selected for on the lidar footprints with the hyper-parameters selected for the footprints derived from satellite imagery on the bottom panel. Please note that experiments I and II were only done on lidar imagery and thus, they only populate the top panel associated with lidar imagery.
